# Evaluation of patient health outcomes of a student-run free clinic in East Harlem

**DOI:** 10.1186/s12909-024-05070-5

**Published:** 2024-03-21

**Authors:** Joy J. Jiang, Katie Link, George Mellgard, Francesca Silvestri, Daniel Qian, Susmita Chennareddy, Michelle Tran, Yoni Goldstein, Gabriela Frid, Isabelle Band, Alexandra Saali, David C. Thomas, Harish Jasti, Yasmin S. Meah

**Affiliations:** Icahn School of Medicine at Mount Sinai, 1468 Madison Avenue, Annenberg Building, 18th Floor Room 18-16, New York, NY 10029 USA

**Keywords:** HEDIS, Free clinic, Student-run, Quality improvement

## Abstract

**Background:**

Most United States medical schools have affiliated student-run free clinics, but the quality of services provided in such contexts compared to national metrics is unknown. This study determines whether a student-run, attending-supervised free clinic servicing a low-income and minority race patient population in New York City can meet national metrics of care.

**Methods:**

Through chart review from January 1, 2020 to December 31, 2020, patient outcomes and service utilization in the Healthcare Effectiveness Data and Information Set were examined and compared to national rates of patients using Medicaid HMO or Medicare. Patients are ≥ 21 years of age, residents of East Harlem, and ineligible for health insurance because of legal residency requirements. The majority identify as Hispanic and speak Spanish as their primary language. All patients who were seen in the clinic during the 2020 calendar year were included. The primary study outcome is the number of Healthcare Effectiveness Data and Information Set measures in which patients, seen in a student-run free clinic, meet or exceed national comparisons.

**Results:**

The healthcare outcomes of 238 patients, mean age 47.8 years and 54.6% female, were examined in 18 Healthcare Effectiveness Data and Information Set measures. The student-run free clinic met or exceeded national metrics in 16 out of 18 categories.

**Conclusions:**

The student-run free clinic met or exceeded the national standard of care according to national metrics. Evidence-based priorities have been clarified for future improvement. Other student-run free clinics should similarly evaluate the quality of their services.

**Supplementary Information:**

The online version contains supplementary material available at 10.1186/s12909-024-05070-5.

## Background

An estimated 27.2 million (8.3%) of individuals in the United States (US) lack any form of health insurance [1]. Uninsured adults have been shown to consistently experience significantly worse health outcomes [2], a trend that has been further exacerbated by the COVID-19 pandemic which has disproportionately impacted the vulnerable populations already experiencing significant healthcare barriers [3].

First developed in the US in the 1960s, student-run free clinics (SRFCs) serve as critical safety nets that amplify medical services critical to the well-being of the uninsured who may otherwise receive healthcare exclusively through urgent care and emergent care settings [4, 5]. Health professions students volunteer at the helm of these clinics, both providing care for patients with supervision from faculty physicians and leading the executive organization of clinic operations. Today, over 75% of institutions of the US Association of American Medical College have SRFCs, offering a wide range of services in addition to primary care such as laboratory evaluations, medication provisions, psychiatric and specialty care, and social services [6, 7].

Positioned at the intersection of advocacy, education, medical care, and volunteerism [4, 7], SRFCs remain subject to ethical debate about the quality of services provided to marginalized populations. For example, SRFCs permit inexperienced student volunteers the opportunity to train at the expense of patients who have no other options [8], raising the concern for possible sub-standard care [9, 10]. In addition to novice clinical insight or lack of financial obligation to patients, SRFCs encounter additional unique challenges such as annual student leadership turnover, limited operating budget, and insufficient volunteer supervising physicians [7]. Despite these concerns, previous work has revealed that SRFCs can deliver services that equal those of typical provider-led healthcare facilities [7, 11–17]. However, the quality of primary care provided by SRFCs has yet to be compared to national metrics as outlined by the Healthcare Effectiveness Data and Information Set (HEDIS) established by the National Committee on Quality Assurance.

We sought to quantitatively assess how our SRFC, the East Harlem Health Outreach Partnership (EHHOP), compares to the national standards of primary care. Previously, the Mental Health Clinic of EHHOP evaluated their mental healthcare services with the HEDIS performance metrics and ascertained that their services were comparable or superior to these standards [8, 12]. Our primary goals were to (1) analyze demographics and morbidities of EHHOP’s patient population and (2) compare outcomes at EHHOP to national HEDIS metrics. Understanding and ensuring that student-run free clinics meet national standards are essential for delivering high-quality, safe, and effective healthcare services and securing the resources and support necessary for clinics’ improvements and sustainability.

## Methods

### Study population

Founded in 2004, EHHOP is the student-run, attending-directed clinic of the Icahn School of Medicine at Mount Sinai. EHHOP is situated in East Harlem, which is comprised of 43% Hispanic and 29% Black residents as of 2019 data; 31.2% of people live below the poverty line, more than double the overall rate in New York [18]. EHHOP provides primary care and social services, as well as specialized care in cardiology, mental health, ophthalmology, podiatry, and women’s health, at no out-of-pocket cost to residents of East Harlem who are over 21 years of age and ineligible for health insurance due to legal residency requirements. Those who are eligible for insurance are phased into the healthcare system through a facilitated-benefits process guided by students and on-staff social workers. Patients at EHHOP have access to social services, medications, and radiographic services at no fee; philanthropic funds and grants cover the bulk of these services.

We included all patients who had at least one appointment at EHHOP’s primary care clinic, whether in-person or telehealth, between January 1, 2020 and December 31, 2020. We selected 2020 because this year not only represents the most recent complete year of data at time of analysis, but also captures the impact of COVID-19 on clinic operations. While many free clinics shuttered across the country, EHHOP continued services through telehealth from March through September 2020, with select in-person appointments, and officially adopted a hybrid telehealth and live model thereafter. This study was approved by Mount Sinai IRB #2100807

### Data collection

Patient data were collected through Slicer Dicer, a tool for data exploration in the Epic electronic health record system, and manual chart review for the 2020 calendar year by all authors for each metric. Morbidities were ascertained by ICD-10 codes [19, 20]. A comprehensive list of ICD-10 codes is included in Supplementary Table 1. In describing clinic population outcomes, national rates for comparison were selected based on most recent availability. 2020 was the intended year for national rates to be compared against EHHOP rates, as the EHHOP patient outcomes were collected for the year 2020. However, if the rate was not available for 2020, we included the statistic for the most recent year thereafter.

### Evaluation of clinic performance

Released by the National Committee for Quality Assurance (NCQA) Healthcare, a major non-profit organization in healthcare accreditation, the HEDIS is a tool used to measure performance metrics in healthcare where improvements can make a meaningful difference in patient care. HEDIS allows the assessment of domains that include the effectiveness of care, access/ availability of care, healthcare utilization, and measures reported in the electronic medical record. Licensing from the NCQA is not needed to use their data.

We compare EHHOP outcomes to those published under HEDIS. We have maintained full fidelity to the description of the metric, as shared on the HEDIS website. To determine adherence to screening guidelines examined in this study, frequency and timing intervals derived from HEDIS were implemented. These metrics were chosen according to their applicability to our patient population. Patients at EHHOP are greater than 21 years of age and typically less than 65 years of age. At 65 years of age, most patients, with some exceptions, are eligible for Medicare and transferred accordingly out of the clinic. As a result, we excluded metrics for children or adolescents as well as most metrics for older adults greater than 65 years of age. Two exceptions were made for vaccination for flu and pneumococcal and osteoporosis screening for older women. We included these metrics because EHHOP has robust vaccination and osteoporosis screening programs. Then, other metrics were not included because of the relative sparse occurrences of these conditions or events. Finally, because the Mental Health Clinic of EHHOP has previously examined their services against the HEDIS performance metrics, measures pertaining to psychiatric care were not re-examined.

Most national data used for comparison against metrics derived from the clinic were collected from the NCQA HEDIS website [21]. Among the publicly available metrics, the benchmark population of the national Medicaid Health Maintenance Organization (HMO), a program intended for low-income citizens and legal residents [22], was deemed to most resemble the EHHOP patient population in terms of socioeconomic status. When Medicaid rates were not available for comparison for specific measures according to the HEDIS website, the Medicare population was selected to represent the next best population with non-private insurance. We note that the Medicare population is much less comparable to the clinic population because its patients are primarily older adults and people with disabilities.

### Statistical analysis

The statsmodels 0.14.0 package in Python was used for statistical analysis. *p* < 0.05 was considered statistically significant. The pwr.p.test 1.3 package in R was used for power and effect size analyses.

## Results

In 2020, 238 patients were seen in EHHOP clinic (Table 1). The mean age was 47.8 years (95% CI [confidence interval] 46.3 – 49.3). 130 patients identified as female (54.6%), and Spanish was the predominant primary language for 185 patients (77.7%). The top ethnic backgrounds were Mexican (48.2%, 67/238), non-Hispanic (13.7%, 19/238), Latin American (12.9%, 18/238), Dominican (6.5%, 9/238), and Puerto Rican (6.5%, 9/238). Among the 91 patients with recorded educational level, 11 did not receive formal education (12.1%), and 48 patients attained education to the 1st – 8th grade level (52.7%), 24 patients to high school (27.4%), and 8 to graduate school (8.8%). 99 patients were employed (99/188, 52.7%), and 117 experienced food insecurity (61.9%, 117/189). 111 patients received assistance in access to social services only (46.6%, 111/238), 187 patients (78.6%, 187/238) received social services assistance, and 51 did not use social services (21.4%, 51/238). During the COVID-19 pandemic, 148 patients (62.2%, 148/238) received grant assistance.

**Table 1:** Demographic characteristics and HEDIS metrics among East Harlem residents who attended a student run free clinic (EHHOP), New York City, 2020–2021, *N* = 238

Demographic	Patients (%)
**Mean age (years)**	47.8
**Female sex**	130 (54.6%)
**Language**	
Spanish	185 (77.7%)
English	52 (21.8%)
Albanian	1 (0.4%)
**Ethnic background**	
Mexican	67 (28.2%)
Non-Hispanic	19 (7.9%)
Latin American	18 (7.6%)
Dominican	9 (3.8%)
Puerto Rican	9 (3.8%)
Missing data/ unknown	116 (48.7%)
**HEDIS Metric**	
Food insecurity assessment	
Food insecure	117 (49.2%)
Not food insecure	72 (30.2%)
Missing data/ unknown	49 (20.6%)
Social services utilization	187 (78.6%)
Grant assistance received	148 (62.2%)
Employment status assessment	
Employed	99 (41.6%)
Not employed	60 (25.2%)
Missing data/ unknown	79 (33.2%)
Education level assessment	
Between 1st and 8th grade	48 (20.2%)
High school	24 (10.1%)
Graduate	8 (3.5%)
None	11 (4.6%)
Missing data/ unknown	147 (61.8%)

**Fig. 1:**
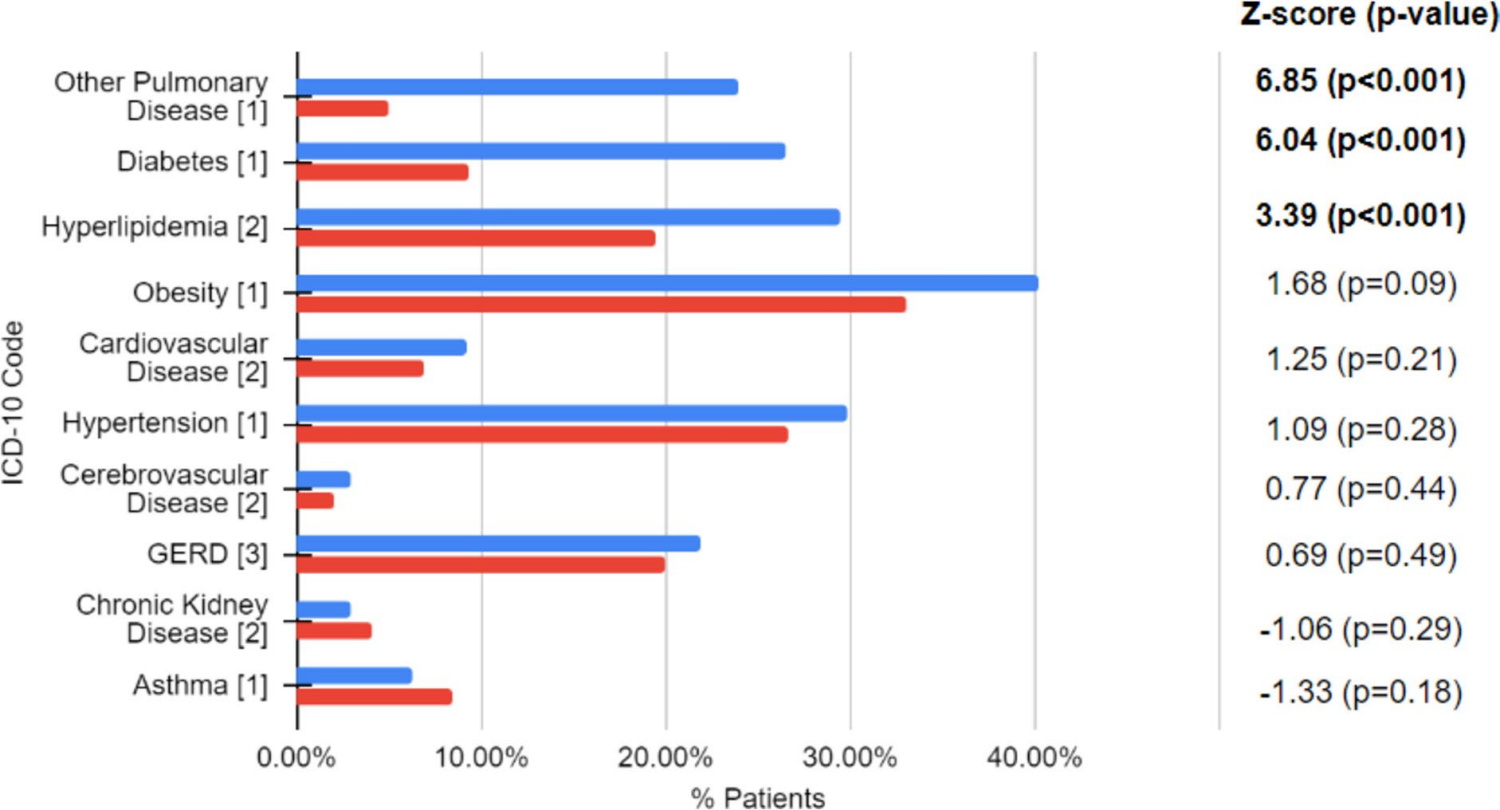
Comparison of morbidities of patients at the East Harlem Health Outreach Partnership clinic to national rates. Legend: Blue indicates 2020 EHHOP outcomes, and red indicates national rates. Numbers in brackets signify data source for national rate: [1] Center for Disease Control 2020, [2] National Ambulatory Medical Care Survey 2018, [3] National Institute for Diabetes and Digestive and Kidney Disease 2004. Significant 1-proportion z-scores are bolded

Among these 238 patients, the most prevalent morbidities were hypertension (29.8%, 71/238), hyperlipidemia (29.4%, 70/238), type 2 diabetes (26.5%, 63/238), and non-asthma pulmonary disease (i.e. ICD-10 codes J00 – J99 with the exception of J45) (24.0%, 57/238). In comparison to national statistics, patients of EHHOP experienced higher rates of non-asthma pulmonary disease, diabetes, and hyperlipidemia (Fig. 1). Power analyses to measure true statistical difference between EHHOP and national rates, as well as Cohen’s h to quantify the magnitude of effect size confirmed statistical significance (Table 2). ICD-10 codes corresponding to these diseases can be found in Supplementary Table 1.

**Table 2:** Power and Cohen’s d for effect size for patient morbidities

ICD-10 Code	Power	Cohen's h
Other Pulmonary Disease	1	0.616
Diabetes	0.999	0.462
Hyperlipidemia	0.95	0.234
Obesity (BMI > = 30)	0.405	0.15
Cardiovascular Disease	0.264	0.861
Asthma	0.238	0.081
Chronic Kidney Disease	0.164	0.631
Cerebrovascular Disease	0.132	0.054
GERD	0.111	0.469
Hypertension	0.071	0.195

**Fig. 2:**
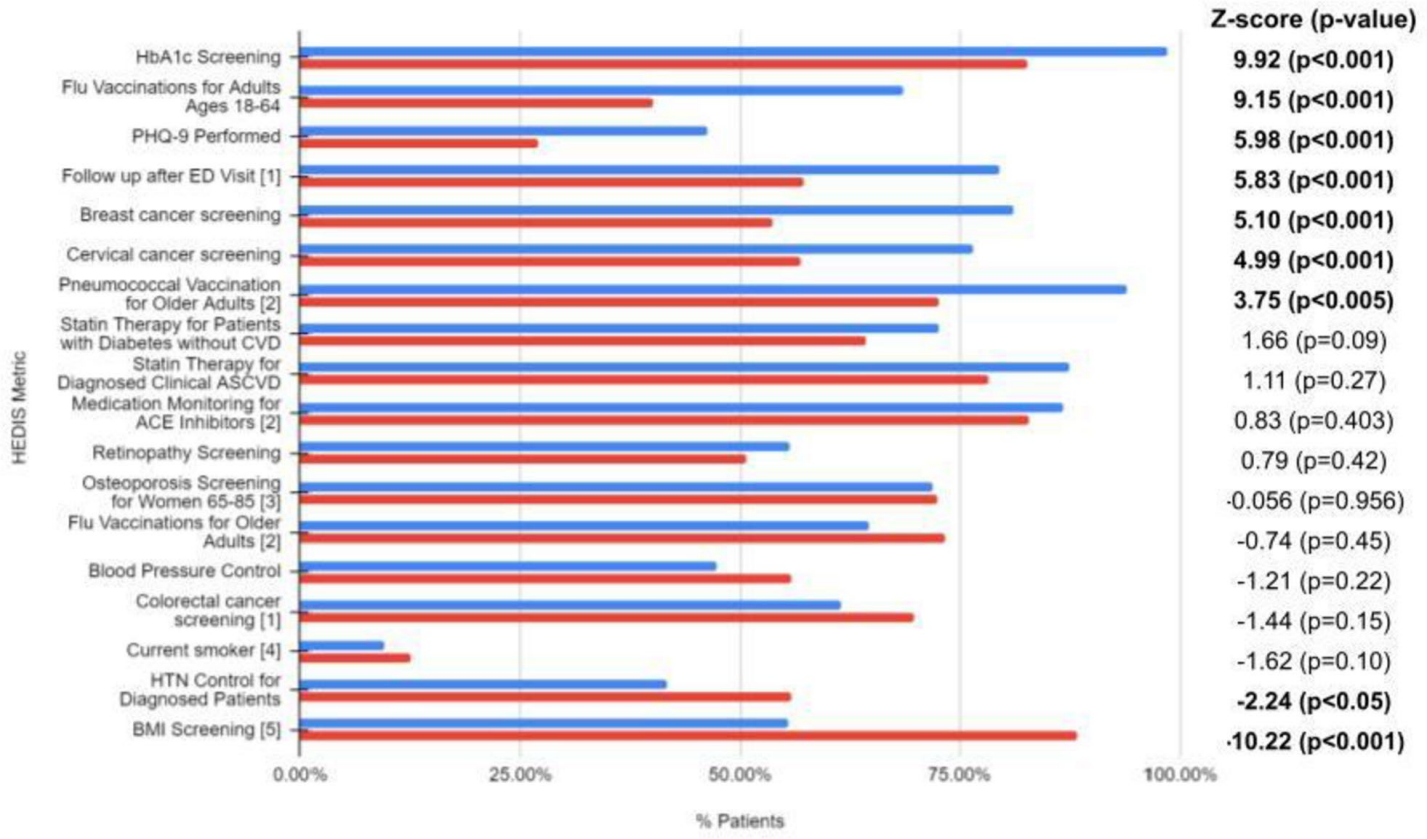
Comparison of HEDIS patient management outcomes to national rates. Legend: Blue indicates 2020 EHHOP outcomes, and red indicates national rates. National rates are derived from Medicaid 2020, unless otherwise signified by numbers in brackets: [1] Medicare HMO 2020, [2] Medicare HMO 2018, [3] Medicare HMO 2017, [4] Centers for Disease Control 2020, [5] Medicaid HMO 2019. Significant 1-proportion z-scores are bolded

Notably, EHHOP excelled in several metrics of health screening (Fig. 2; Table 3). In comparison to those on Medicaid HMO in 2020, EHHOP patients experienced significantly higher rates of HbA1C screening (98.4%, 62/63), higher rates of breast cancer screening (81.1%, 43/53), and higher rates of cervical cancer screening (76.5%, 88/115). In addition, compared to those on Medicaid HMO in 2020, a significantly higher proportion of patients were screened for depression using the PHQ-9 (46.2%, 110/238 vs 26.9%).

**Table 3:** Power and Cohen’s d for effect size for HEDIS metrics

HEDIS Metric Description	Power	Cohen's h
Flu Vaccinations for Adults Ages 18–64	1	0.582
Retinopathy Screening	1	0.765
BMI Screening	1	0.765
PHQ-9 Performed	0.999	0.404
Follow up after ED Visit	0.999	0.487
HbA1c Screening	0.998	0.602
Cervical cancer screening	0.996	0.434
Breast cancer screening	0.992	0.597
HTN Control for Diagnosed Patients	0.598	0.285
Statin Therapy for Diagnosed Clinical ASCVD	0.331	0.01
Current smoker	0.331	0.01
Statin Therapy for Patients with Diabetes without CVD	0.316	0.177
Colorectal cancer screening	0.316	0.177
Pneumococcal Vaccination for Older Adults	0.179	0.17
Blood Pressure Control	0.179	0.17
Medication Monitoring for ACE Inhibitors	0.125	0.109
Flu Vaccinations for Older Adults	0.12	0.186
Osteoporosis Screening for Women 65–85	0.05	0.0112

EHHOP also performed well in subjects pertaining to maintenance and monitoring. 20 patients were hospitalized in 2020 (8.4%, 20/238), and 60 visited the emergency department (25.2%, 60/238). Among the 60 patients visited the ED, there were 112 unique visits. Patients at EHHOP received higher rates of follow-up after ED visit (79.5%, 89/112) in comparison to those using Medicare HMO (57.2%). Moreover, among adults 18 – 64 years of age, a higher proportion of EHHOP patients received flu vaccination compared to those on Medicaid HMO in 2020 (68.6%, 151/220 vs 40.0%). Among the 17 adults > 65 years of age, 16 patients have pneumococcal vaccination compared to those on Medicare HMO in 2018 (94.1%, 16/17 vs 72.7%).

However, EHHOP performed worse than national rates in a few key areas. Body mass index (BMI) screening at EHHOP was 55.5% (132/238) in comparison to the 2019 Medicaid HMO rate of 88.4%. This could be attributed to the fact that weight is not taken at every visit. The rate of hypertension control to < 140/90 mm Hg for diagnosed patients (41.7%, 25/60) was also lower than that of 2020 Medicaid HMO (55.9%).

In all, we found that EHHOP performed statistically better than or equivalent to national data in 16 out of 18 outcomes. However, EHHOP performed statistically worse in 2 out of 18 outcomes, namely BMI screening and hypertension control.

## Discussion

Overall, EHHOP has been meeting or exceeding the standard of care compared to that provided to Medicaid patients in the majority of examined HEDIS metrics. This is notable given our patients are severely disenfranchised financially, face food insecurities, and inability to access federal and state programming due to residency status [12]. As a result, compared to the national average, a larger portion of the 2020 patient population suffered from illnesses such as non-asthma pulmonary disease, diabetes, and hyperlipidemia.

In spite of these challenges, EHHOP was able to perform at a similar level of care provided to insured patients who receive care from hospital systems. Because of the observational nature of this study and lack of a comparison group, we cannot causally link outcomes to or definitively attribute associations found with any specific features of the clinic. However, we hypothesize that increased time that medical students are able to spend with their patients, despite lack of experience, lead to comprehensive provision of increased preventive measures and patient follow-up. Each student sees no more than two patients in one clinic day. Moreover, each patient visits the clinic every 1–3 months and rarely goes without contact by the clinic for more than 6 months. EHHOP has identified patients of greatest medical need, such as those who have two or more chronic morbidities and follows them at a more regular basis, ensuring patients receive timely lab work and appointments every 6 months.

There are several important limitations to this study. Major limitations include that fact that first, this study was performed on a single site and thereby cannot generalize to other US free clinics. Second, due to inconsistent reporting in the 2020 Medicaid HMO data by NCQA, it was necessary to extract metrics from the subsequent year or to make comparisons to other insurance plans. In the few cases in which the Medicare population was used as the national metric, we note that Medicare is less similar to the patient population in comparison to Medicaid because the Medicare population is comprised of primarily older adults and people with disabilities. We nevertheless decided to benchmark against Medicare because it provides a basis for comparison to a population with national non-private insurance. Third, because patients at EHHOP are largely Hispanic, the Hispanic paradox could bias results [23]. The Hispanic paradox describes an empiric phenomenon in which people who are Hispanic may have significantly better health outcomes in comparison to other minority groups because of selection bias, in which those who immigrate are more likely to be young and healthy, and because of a “reverse salmon” effect, in which those who persons who fall ill are more likely to return home for palliative care. We are unable to quantify the impacts of this bias, as it occurs on a population level as opposed to an individual basis. As this study includes the comparison of certain health conditions between the majority Hispanic EHHOP population and a less Hispanic national population, there could be the potential that patients in our clinic are recorded to have better control of some health morbidities, such as blood pressure, than they actually do.

Other limitations include the fact that the size and age range of the EHHOP patient population precluded examination of most HEDIS metrics. Moreover, the electronic medical records of patients sometimes offered incomplete information, which could lead to underreporting of conditions and disease management. This issue is not uncommon in healthcare institutions [24, 25]. Specifically, to our study, incomplete electronic medical records may have impacted records of hospitalization and emergency department presentations. Given high healthcare utilization rates during the pandemic during New York City, it is surprising that such low numbers of persons were hospitalized and sought emergency care, and it is possible that some visits were not captured. Despite auditing efforts for data validity, we hypothesize that overflow and triaging hospital in 2020 might have affected the consistency of how these encounters were recorded in the electronic medical record. Moreover, the COVID-19 pandemic had a significant impact on clinic services, including the diminished provision of services and collection of biometrics and laboratory values [26, 27]. Similarly, in 2020, primary care services were also impacted across the nation, which could have potentially affected the outcomes at the Medicare and Medicaid supported facilities used as comparative metrics. Nevertheless, patient management outcomes were equivalent to or higher than national outcomes during 2020 or pre-pandemic time period, reflecting EHHOP’s excellent standard of care.

Nonetheless, the clinic lacks comparatively in a couple of key areas. Incomplete BMI screening indicates the importance of standardizing weight measurements at each in-person visit. Meanwhile, improving hypertension control necessitates investigation into adequate patient follow-up, access to medications, and especially availability of nutritious food. We also note that assessing EHHOP patient outcomes to those using Medicaid HMO may be a fallible comparison as patients with Medicaid may be subject to substandard services due segregated care [28].

In the future, prospective quality improvement directed projects will be aimed at ameliorating these metrics, with a special focus on BMI screening and hypertension management. We will also partake in a more detailed collection of comprehensive social information to determine how these circumstances impact health outcomes. Moreover, we aim to pursue specific comparisons of groups within EHHOP, such as those who have substance use disorders or those who use services at the mental health or women’s health clinics, would enable better, targeted intervention. In addition, we plan to standardize the data collection process undertaken in this study with the objective of providing continuous evidence-based care specific to EHHOP patients, so that we can continue to evaluate clinic services in comparison to HEDIS measures in future years. Finally, we hope that the results of this study encourage other SRFCs to compare their patient outcomes to national metrics.

We believe that the basis of achieving these future targets relies upon cultivating a relationship of trust between SRFCs and their patient populations to provide longitudinal care and thus health improvements [4]. Given the complex past of the exploitation of vulnerable patients from underserved backgrounds, respect for and partnership with the patient must be paramount to enact preventative care and safeguard long-term quality of life [4].

## Conclusions

In conclusion, these findings suggest that EHHOP is meeting or exceeding the national standard of care in the majority of 18 examined HEDIS metrics. This study may indicate the potential of SRFC to provide high quality care comparable to that provided for insured patients. We hope that this project can serve as an example for other SRFCs to critically evaluate the quality of their services.

### Supplementary Information

**Additional file 1: Supplementary Table 1. **ICD-10 codes for comorbidity assessment among patients of the East Harlem health outreach partnership clinic in 2020.

## Data Availability

Datasets generated and/or analyzed during the current study are not publicly available due to the sensitive nature of data pertaining to individuals lacking legal residency requirements but are available from the corresponding author on reasonable request.
