# Supplementary material for: Evaluation of patient health outcomes of a student-run free clinic in East Harlem
Source: BMC Med Educ. 2024 Mar 21;24:323. doi: 10.1186/s12909-024-05070-5 (PMC10958952; doi:10.1186/s12909-024-05070-5)
Supplement: Supplementary file 1 — Additional file 1: Supplementary Table 1. ICD-10 codes for comorbidity assessment among patients of the East Harlem health outreach partnership clinic in 2020. [file 12909_2024_5070_MOESM1_ESM.docx]

**Supplementary Table 1.** ICD-10 Codes for Comorbidity Assessment among Patients of the East Harlem Health Outreach Partnership Clinic in 2020

| **Comorbidity** | **ICD-10 Diagnosis Code** |
| --- | --- |
| **Cardiovascular Disease** | Chronic rheumatic heart diseases: I05 – I09  Hypertensive Heart Disease: I11  Hypertensive heart and chronic kidney disease: I13  Heart Failure: I50  Heart disease, unspecified: I51  Angina Pectoris: I20  Acute Myocardial Infarction: I21  STEMI and NSTEMI MI: I22  Certain current complications following STEMI and NSTEMI MI within 28 days: I23  Other acute ischemic heart diseases: I24  Chronic Ischemic Heart Disease: I25  Acute Pericarditis: I30  Other diseases of pericardium: I31  Pericarditis in diseases classified elsewhere: I32  Acute and subacute endocarditis: I33  Nonrheumatic mitral valve disorders: I34  Nonrheumatic aortic valve disorders: I35  Nonrheumatic tricuspid valve disorders: I36  Endocarditis, valve unspecified: I38  Endocarditis and heart valve disorders: I39  Acute myocarditis: I40  Myocarditis in diseases classified elsewhere: I41   Cardiomyopathy: I42   Cardiomyopathy in diseases classified elsewhere: I43   Atrioventricular and left bundle-branch block: I44   Other conduction disorders: I45   Cardiac arrest: I46   Paroxysmal tachycardia: I47   Atrial fibrillation and flutter: I48   Other cardiac arrhythmias: I49   Complications and ill-defined descriptions of heart disease: I51  Other heart disorders in diseases classified elsewhere: I52  Diseases of arteries, arterioles, and capillaries: I70 – I79  Phlebitis and thrombophlebitis: I80  Varicose veins of lower extremities: I83  Varicose veins of other sites: I86 |
| **Cerebrovascular Disease** | Nontraumatic Subarachnoid Hemorrhage: I60  Nontraumatic Intracerebral Hemorrhage: I61  Other and unspecified nontraumatic intracranial hemorrhage: I62  Cerebral Infarction: I63  Occlusion and stenosis of precerebral arteries, not resulting in cerebral infarction: I65  Occlusion and stenosis of cerebral arteries, not resulting in cerebral infarction: I66  Other cerebrovascular diseases: I67  Cerebrovascular disorders in diseases classified elsewhere: I68  Sequelae of cerebrovascular disease: I69 |
| **Diabetes Mellitus** | Encounter for Screening for Diabetes Mellitus: Z13.1  Drug or chemical induced diabetes mellitus: E09  Type 1 Diabetes Mellitus: E10  Type 2 Diabetes Mellitus: E11  Malnutrition-related Diabetes Mellitus: E12  Other Specified Diabetes Mellitus: E13  Unspecified Diabetes Mellitus: E14 |
| **Gastrointestinal Disease** | **GERD:** K21  **Dyspepsia:** K30  **Other GI Disease:** K00 – K95 with the exception of K21 and K30 |
| **Hyperlipidemia** | E78 |
| **Hypertension** | Essential (primary) Hypertension: I10  Hypertensive Heart Disease: I11  Hypertensive Chronic Kidney Disease: I12  Hypertensive heart and chronic kidney disease: I13  Secondary Hypertension: I15  Hypertensive Crisis: I16 |
| **Pulmonary Disease** | **Asthma:** J45  **COPD:**  Simple and Mucopurulent Chronic Bronchitis: J41  Unspecified Chronic Bronchitis: J42  Emphysema: J43  Other COPD: J44  **Other Pulmonary Disease:** J00 – J99 with the exception of J45 |
